# Social determinants of obesity in American Indian and Alaska Native peoples aged ≥ 50 years

**DOI:** 10.1017/S1368980022000945

**Published:** 2022-08

**Authors:** R Turner Goins, Cheryl Conway, Margaret Reid, Luohua Jiang, Jenny Chang, Kimberly R Huyser, Angela G Brega, John F Steiner, Amber L Fyfe-Johnson, Michelle Johnson-Jennings, Vanessa Hiratsuka, Spero M Manson, Joan O’Connell

**Affiliations:** 1 Department of Social Work, Western Carolina University, College of Health and Human Sciences, 3971 Little Savannah Road, Cullowhee, NC 28723, USA; 2 Quality Management, Veterans Health Administration, Asheville, NC, USA; 3 Department of Health Systems, Management & Policy, Colorado School of Public Health, University of Colorado, Denver, CO, USA; 4 Department of Epidemiology, University of California Irvine, Irvine, CA, USA; 5 Department of Sociology, University of British Columbia, Vancouver, BC, Canada; 6 Department of Community & Behavioral Health, Colorado School of Public Health, University of Colorado, Denver, CO, USA; 7 Institute for Health Research, Kaiser Permanente Colorado, Denver, CO, USA; 8 Elson S. Floyd College of Medicine, Washington State University, Pullman, WA, USA; 9 Department of Indigenous Studies, University of Saskatchewan, Saskatoon, SK, Canada; 10 Center for Human Development, University of Alaska Anchorage, Anchorage, AK, USA

**Keywords:** American Indian peoples, Alaska Native peoples, Obesity, Social determinants of health, Older adults

## Abstract

**Objective:**

American Indian and Alaska Native peoples (AI/AN) have a disproportionately high rate of obesity, but little is known about the social determinants of obesity among older AI/AN. Thus, our study assessed social determinants of obesity in AI/AN aged ≥ 50 years.

**Design:**

We conducted a cross-sectional analysis using multivariate generalised linear mixed models to identify social determinants associated with the risk of being classified as obese (BMI ≥ 30·0 kg/m^2^). Analyses were conducted for the total study population and stratified by median county poverty level.

**Setting:**

Indian Health Service (IHS) data for AI/AN who used IHS services in FY2013.

**Participants:**

Totally, 27 696 AI/AN aged ≥ 50 years without diabetes.

**Results:**

Mean BMI was 29·8 ± 6·6 with 43 % classified as obese. Women were more likely to be obese than men, and younger ages were associated with higher obesity risk. While having Medicaid coverage was associated with lower odds of obesity, private health insurance was associated with higher odds. Living in areas with lower rates of educational attainment and longer drive times to primary care services were associated with higher odds of obesity. Those who lived in a county where a larger percentage of people had low access to a grocery store were significantly less likely to be obese.

**Conclusions:**

Our findings contribute to the understanding of social determinants of obesity among older AI/AN and highlight the need to investigate AI/AN obesity, including longitudinal studies with a life course perspective to further examine social determinants of obesity in older AI/AN.

Worldwide, the prevalence of obesity, defined as abnormal or excessive fat accumulation, has almost tripled since 1975^(1)^. Age-adjusted prevalence of obesity in the US adult population was 42 % in 2017–2018^(2)^. By 2030, it is predicted that 51 % of the US population will be obese^(3)^. Obesity is associated with many health complications and diseases and contributes substantially to national health care costs. Indeed, medical costs for obesity-related illness in adults were estimated to be as high as $209·7 billion in 2008, with 20·6 % of the US national health expenditures treating obesity-related conditions^(4)^.

Although rates of obesity are increasing for all racial and ethnic groups, there are significant disparities among them. In 2018, 48 % of American Indian and Alaska Native peoples (AI/AN) aged ≥ 18 years were obese compared to 31 % of non-Hispanic Whites^(5)^. Many theorised causes of racial and ethnic disparities in obesity are not fully understood such as the role of historical trauma^(6)^, but differences in social and economic advantages play a key role^(7)^. Social determinants of health are multifactorial and include the conditions in the environments where individuals are born, live, work, play, and age. Examples of social determinants examined in the literature have included health care access, housing and transportation resources, nutritious food access, education, socio-economic status, ethnic and cultural identity, sex, and neighbourhood safety and facilities^(8)^. Such social determinants can have serious implications, especially for the effective management of chronic health conditions such as obesity.

Relative to other racial and ethnic populations, little is known about the specific influence of social determinants of health among Native peoples. The social, political and historical context of AI/AN is complex. An ecological model proposed for understanding obesity in Aboriginal children in Canada appears applicable to older AI/AN^(9)^. In this model, six concentric circles characterise the mutual, reciprocating effects of social determinants of health. The outer circle represents historical factors, and moving inwards, followed by society, built environment, community, sociocultural environments, interpersonal and individual factors. Poverty, substandard housing, limited built environment, geographic isolation and health care access are especially powerful influences among AI/AN.

A recent, systematic review revealed that no published studies have specifically examined social determinants of obesity among older AI/AN and identified only three publications in the past decade that included adult samples^(10)^. Thus, our study sought to redress this imbalance by examining the association of social determinants of obesity among AI/AN aged ≥ 50 years. Often research on older adults defines their samples as ≥ 65 years of age. However, given the earlier onset of chronic conditions such as obesity as well as shorter life expectancies among AI/AN^(11)^, we dropped the age range for this study to aged ≥ 50 years.

For this study, we excluded persons with diabetes for two reasons. First, obesity is not always considered a negative outcome among older adults with diabetes. That is, some research has shown that obesity is associated with favourable health outcomes in this group, which is called ‘obesity paradox’ in a rich body of literature^(12)^. Thus, the relationships between social determinants and obesity among adults with diabetes are complicated and might be hard to explain, especially using cross-sectional data. Second, for people with diabetes, treatment goals usually include weight loss through pharmacological and/or health behaviour interventions^(13)^. This further complicates the relationship between social determinants and obesity among those with diabetes. Yet, given that obesity increases the incidence of diabetes across the life course, research in older AI/AN without diabetes can provide important insight into social and environmental factors that may place older AI/AN at risk for developing diabetes.

## Methods

### Data

Approximately 30 % of AI/AN obtain health care through the Indian Health Service (IHS), a federal agency charged with providing health care to members of federally recognised tribes and tribal entities in the USA. The IHS system includes hospitals, clinics and health programmes operated by the federal government, tribal organisations, and urban Indian health programmes, and they known collectively as I/T/Us and serve approximately 2·56 million AI/AN^(14)^.

Our study drew upon data from the IHS *Improving Health Care Delivery Data Project* (IHS Data Project), which houses health status, service use and cost data for over 640 000 AI/AN, representing nearly 30 % of those who use IHS services^(15)^. The IHS Data Project was developed to provide IHS, tribal leaders and AI/AN communities with information about the health needs of AI/AN with chronic diseases to aid in identifying and prioritising strategies to improve health outcomes and resource allocation.

The IHS Data Project includes data from a purposeful sample of AI/AN living in the geographic areas of 15 IHS Service Units. A Service Unit is an administrative entity either operated by the IHS or a tribe that has responsibility of the IHS programme serving a specific geographic area and hereafter are referred to as ‘project sites’. One project site is located in the Eastern United States, four are in the Northern Plains, two are in the Southern Plains, five are in the Southwest, two are in the Pacific Coast and one is in Alaska. The IHS Data Project developed a data infrastructure that synthesises electronic health record data from multiple IHS platforms from FY2007 to FY2013. The data infrastructure includes demographic, health coverage and service use data from the National Data Warehouse as well as service use data from the Purchased and Referred Care programme. The IHS Data Project sample is comparable to the national IHS service population with respect to age and sex. More information about the assembly of the data infrastructure can be found elsewhere^(15)^.

### Study sample

Our study sample included AI/AN aged ≥ 50 years who used IHS/tribal services in FY2013 and did not have a transplant, end-stage renal disease or malignant cancer as these conditions substantially influence treatment needs; 80 140 users met these criteria. We then sequentially excluded patients who were missing county-level measures of social determinants of interest (*n* 13, from three counties that had too few AI/AN respondents and American Community Survey (ACS) data for AI/AN-specific variables were unavailable), were diagnosed with diabetes (*n* 31 631), had missing or a biologically implausible height, weight or BMI data (*n* 20 731) or lived in a county with < 5 obese study participants (to avoid convergence issues of multilevel models due to sparse clusters, *n* 69). We evaluated biologically implausible height, weight and BMI values in several steps based on prior work. First, we removed extreme height (< 49” or > 84”) and weight (< 75 lbs or > 600 lbs) values^(16)^. We then matched remaining height and weight values by date of service, calculated BMI as weight in kilograms divided by height in metres squared and removed extreme values (<12 or >70)^(17)^. Our final analytic sample included 27 696 adults.

### Measures

To define obesity, we used the Centers for Disease Control and Prevention’s standard BMI categories for adults, which are underweight (< 18·5 kg/m^2^), normal weight (18·5–24·9 kg/m^2^), overweight (25·0–29·9 kg/m^2^) and obese (≥ 30·0 kg/m^2^). We also examined the three subcategories of obesity, which are obese I (30·0–34·9 kg/m^2^), obese II (35·0–39·9 kg/m^2^) and obese III (≥ 40·0 kg/m^2^)^(18)^.

We organised the social determinants measures pursuant to the Healthy People 2020 social determinants of health framework^(19)^, with the following four groupings: (1) health care coverage and access; (2) education and economic stability; (3) neighbourhood and built environment; and (4) social and community context.

We included two measures of health care coverage and access. We measured individual-level health coverage using the following categories: Medicare, Medicaid, private insurance and no coverage other than access to IHS services (yes/no) during FY2013. Health care access was measured by drive time to IHS facilities providing primary care and was categorised as either < 30 min or ≥ 30 min. As has been done in prior research, we used the geographic coordinates for the communities in which the IHS Data Project population lived and the nearest IHS health facilities to estimate community-specific patient drive times^(20)^.

Measures of education and economic stability included county-level educational attainment, household income, households with incomplete kitchen facilities and households with no vehicle access. For each measure, we calculated the median value among the seventy-two counties included in our data and created a dichotomous variable that indicated whether each county was above or below the median value.

The education and income measures were derived from the 2010–2014 ACS special tabulation from the US Census Bureau^(21)^ for people who reported being AI/AN alone or in combination with one or more other race(s) and people who reported having access to IHS services. The educational attainment measure was the percentage of adults aged ≥ 25 years who did not complete high school (median value = 46·0 %). We defined households with a low income as those with incomes below the federal poverty level (FPL). Among the seventy-two counties in our study, the median proportion of households below the FPL was 27·9 %. Thus, for half of the counties, the percentage of households below the FPL was < 27·9 %, and we refer to these counties as lower-poverty counties. The other half of the counties had ≥ 27·9 % of households below the FPL, and we refer to these counties as higher-poverty counties. From the 2000 US Census^(22)^, we obtained data on percentage of households with incomplete kitchen facilities by county (median = 1·8 %) and percentage of households with no vehicle access by county (median = 12·9 %).

Neighbourhood and built environment measures included access to a grocery store and the county’s rural designation. Referring to the USDA Food Environment Atlas^(23)^, we used the percent of the population with low access to a grocery store in 2015. Low access was measured as the percentage of people in a county living > 1 mile from a supermarket or large grocery store if in an urban area, or > 10 miles from a supermarket or large grocery store if in a rural area. The median across counties was 25·3 %. For rurality, we coded counties as urban or rural using the National Center for Health Statistics classifications^(24)^.

Social and community context was measured as the percentage of AI/AN who identified as AI/AN alone or in combination with one or more other race(s). From the 2010 US Census^(25)^, we estimated the percent of the county residents who identified as AI/AN; the median was 14·6 %.

### Statistical analyses

Obesity prevalence as well as individual- and county-level characteristics were summarised by frequencies and percentages for the overall study population and two subpopulations stratified by median county poverty level. Multivariable generalised linear mixed models with a logit link were used to examine the association between obesity and each social determinant with county-level random effects. Each model included all independent variables, and each variable was adjusted by all the other independent variables in the same model. We fit the models for the total study population as well as two subpopulations stratified by county poverty (i.e. higher- and lower-poverty counties). We did not include Medicare coverage in our main analysis since the vast majority of adults aged ≥ 65 years had Medicare coverage. However, we conducted a sensitivity analysis that adjusted for Medicare coverage among adults aged < 65 years. All statistical analyses and variable construction were performed using SAS 9.4 (SAS Institute).

## Results

Table 1 presents the characteristics of the overall study population and the two subpopulations. The mean age of our study population was 61·8 ± 9·6 years (data not shown); 59·9 % were female. Compared to lower-poverty counties where ≤ 27·9 % of households were classified as having incomes below the FPL, the higher-poverty counties had a greater percentage of individuals in the youngest age group of 50–59 years (56·3 % *v*. 50·2 %, respectively).

**Table 1:** Characteristics of study population

	All sample		Lower-poverty counties		Higher-poverty counties		Obesity	
	*n*	%	*n*	%	*n*	%	*n*	%
All sample	27 696	100·0	15 986	57·7	11 710	42·3	11 922	43·0
Demographics								
Sex								
Male	11 119	40·1	6482	40·5	4637	39·6	4658	41·9
Female	16 577	59·9	9504	59·5	7073	60·4	7264	43·8
Age group								
50–59 years	14 615	52·8	8027	50·2	6588	56·3	7093	48·5
60–69 years	7765	28·0	4746	29·7	3019	25·8	3368	43·4
70–79 years	3688	13·3	2228	13·9	1460	12·5	1186	32·2
≥80 years	1628	5·9	985	6·2	643	5·5	275	16·9
Health care coverage and access								
Medicaid coverage								
No Medicaid	25 605	92·5	15 341	96·0	10 264	87·7	11 037	43·1
Medicaid	2091	7·5	645	4·0	1446	12·3	885	42·3
Private insurance								
No private insurance	22 898	82·7	12 837	80·3	10 061	85·9	9645	42·1
Private insurance	4798	17·3	3149	19·7	1649	14·1	2277	47·5
Medicare coverage*								
No Medicare coverage or age ≥ 65 years	25 599	92·4	14 668	91·8	10 931	93·3	10 894	42·6
Medicare coverage and age < 65 years	2097	7·6	1318	8·2	779	6·7	1028	49·0
Other health coverage category								
No additional health coverage other than access to IHS services	12 972	46·8	7183	44·9	5789	49·4	6015	46·4
Had health coverage	14 724	53·2	8803	55·1	5921	50·6	5907	40·1
Drive time to primary care								
<30 min	24 957	90·1	15 177	94·9	9780	83·5	10 679	42·8
≥30 min	2739	9·9	809	5·1	1930	16·5	1243	45·4
Education and economic stability								
County-level educational attainment, ACS 2010–2014: % < high school†								
Counties below median (≤ 46·0 %) (higher education)	16 538	59·7	10 796	67·5	5742	49·0	7021	42·5
Counties above median (> 46·0 %) (lower education)	11 158	40·3	5190	32·5	5968	51·0	4901	43·9
County-level household income: % < 100 % FPL†								
Counties below median (≤ 27·9 %) (lower poverty)	15 986	57·7					6682	41·8
Counties above median (> 27·9 %) (higher poverty)	11 710	42·3					5240	44·7
County-level AI/AN households with incomplete kitchen facilities‡								
Counties below median (≤ 1·8 %)	17 390	62·8	12 955	81·0	4435	37·9	7466	42·9
Counties above median (>1·8 %)	10 306	37·2	3031	19·0	7275	62·1	4456	43·2
County-level AI/AN households with no vehicle access, Census 2000‡								
Counties below median (≤ 12·9 %)	18 887	68·2	14 891	93·2	3996	34·1	7991	42·3
Counties above median (>12·9 %)	8809	31·8	1095	6·8	7714	65·9	3931	44·6
Neighbourhood and built environment								
County-level low access to a grocery store, USDA 2010§								
Counties below median (≤ 25·3 %)	17 205	62·1	10 248	64·1	6957	59·4	7611	44·2
Counties above median (>25·3 %)	10 491	37·9	5738	35·9	4753	40·6	4311	41·1
NCHS 2013 urban–rural indicator								
Non-rural	18 311	66·1	11 874	74·3	6437	55·0	8004	43·7
Rural	9385	33·9	4112	25·7	5273	45·0	3918	41·7
Social and community context								
Percent AI/AN alone, Census 2010‖								
Counties below median (≤ 14·6 %)	8522	30·8	4079	25·5	4443	37·9	3932	46·1
Counties above median (>14·6 %)	19 174	69·2	11 907	74·5	7267	62·1	7990	41·7

IHS, Indian Health Service; ACS, American Community Survey; FPL, federal poverty level; AI/AN, American Indian and Alaska Native; NCHS, National Centre for Health Statistics.

* Over 90 % of adults aged ≥ 65 years had Medicare coverage.

† Data source: American Community Survey 2010–2014 5-year estimates.

‡ Data source: US Census Bureau 2000.

§ Data source: US Department of Agriculture Food Environment Atlas.

‖ Data source: US Census Bureau 2010.

In our study population, 7·5 % had Medicaid coverage, 17·3 % had private insurance and 46·8 % had no coverage other than access to IHS services. Almost 10 % of our study population lived in communities that had a ≥ 30-min drive time to a primary care facility. Approximately 60 % resided in counties where ≤ 46 % of adults aged ≥ 25 years did not complete high school and 57·7 % resided in lower-poverty counties. Most of the study population lived in counties where ≤ 1·8 % of households had incomplete kitchens and ≤ 12·9 % of households did not have access to a vehicle. Sixty-two per cent resided in counties where ≤ 25·3 % of residents had low grocery store access and 66 % lived in non-rural counties. Close to 70 % of the study population resided in counties where > 14·6 % of persons identified as AI/AN.

Table 1 also shows the prevalence of obesity by individual characteristics. Forty-three per cent were classified as obese (BMI ≥ 30·0 kg/m^2^). Obesity was slightly more prevalent among women than men (43·8 % *v*. 41·9 %, respectively), the younger age group (48·5 % in 50–59 years *v*. 16·9 % in ≥ 80 years), those with private insurance (47·5 % *v*. 42·1 %) and those who live in communities that were far from a primary care facility (45·4 % *v*. 42·8 %). In addition, obesity was more prevalent among people who lived in counties with lower educational attainment, lower income, lower vehicle access, higher access to grocery stores, and lower proportion of AI/AN, and counties that were non-rural.

Table 2 presents the distribution of our study sample across the six different obesity classifications overall and in the two subpopulations based on county-level poverty. For the entire study sample, the mean BMI was 29·8 ± 6·6. There was a greater percentage of obese persons in higher-poverty counties than lower-poverty counties (44·8 % *v*. 41·8 %, respectively).

**Table 2:** BMI of study population

	Entire study sample		Lower-poverty counties		Higher-poverty counties	
	*n*	%	*n*	%	*n*	%
Underweight (< 18·5)	405	1·5	236	1·5	169	1·4
Normal weight (18·5–24·9)	5847	21·1	3609	22·6	2238	19·1
Overweight (25·0–29·9)	9522	34·4	5459	34·2	4063	34·7
Obese I (30·0–34·9)	6919	25·0	3816	23·9	3103	26·5
Obese II (35·0–39·9)	3114	11·2	1806	11·3	1308	11·2
Obese III (≥ 40)	1889	6·8	1069	6·6	829	7·1

Table 3 presents the results of multivariate generalised linear mixed regression analyses. After controlling for age, health care coverage, health care access, education and economic stability indicators, county-level low grocery store access, county rural designation, and the percent of AI/AN alone, females were more likely to be obese than males (OR = 1·09, 95 % CI (1·03, 1·14), *P* < 0·01). The younger age group (50–59 years) was associated with higher risk of obesity, with decreasing OR as age increased. Medicaid insurance was associated with lower odds of obesity (OR = 0·89, 95 % CI (0·81, 0·98), *P* < 0·05), while private insurance (OR = 1·19, 95 % CI (1·11, 1·27), *P* < 0·001) and longer drive time to primary care (OR = 1·12, 95 % CI (1·02, 1·23), *P* < 0·05) were associated with higher odds of obesity. In stratified analyses, the significant association between obesity and age persisted. However, sex- and community-level drive time to primary care were only significantly associated with obesity in higher-poverty counties, while private insurance was only significantly associated with obesity among people in lower-poverty counties.

**Table 3:** Adjusted OR for obesity for study population

	All sample (*n* 27 696)		Lower-poverty counties (*n* 15 986)		Higher-poverty counties (*n* 11 710)	
	OR	95 % CI	OR	95 % CI	OR	95 % CI
Demographics						
Sex						
Male (reference)						
Female	1·09	1·03, 1·14**	1·01	0·94, 1·07	1·21	1·12, 1·31***
Age group						
50–59 years (reference)						
60–69 years	0·81	0·77, 0·86***	0·83	0·77, 0·89***	0·80	0·73, 0·87***
70–79 years	0·51	0·47, 0·55***	0·54	0·49, 0·60***	0·47	0·41, 0·53***
≥ 80 years	0·22	0·19, 0·25***	0·22	0·18, 0·26***	0·22	0·18, 0·28***
Health care coverage and access						
Medicaid coverage	0·89	0·81, 0·98*	0·89	0·75, 1·05	0·91	0·81, 1·03
Private insurance	1·19	1·11, 1·27***	1·28	1·18, 1·39***	1·04	0·93, 1·16
Drive time to primary care ≥ 30 min	1·12	1·02, 1·23*	1·08	0·92, 1·26	1·16	1·04, 1·30**
Education and economic stability						
County-level educational attainment, ACS 2010–2014: % < high school†						
Counties above median	1·20	1·08, 1·34***	1·17	1·05, 1·30**	1·28	1·06, 1·55*
County-level household income: % < 100 % FPL†						
Counties above median	1·03	0·92, 1·15				
County-level AI/AN households with incomplete kitchen facilities‡						
Counties above median	0·98	0·87, 1·12	0·88	0·75, 1·03	1·09	0·89, 1·33
County-level AI/AN households with no vehicle access, Census 2000‡						
Counties above median	0·99	0·88, 1·12	1·09	0·92, 1·28	0·95	0·78, 1·16
Neighbourhood and built environment						
County-level low access to a grocery store, USDA 2010§						
Counties above median	0·87	0·790,·97**	0·94	0·85, 1·04	0·77	0·63, 0·94*
Rural, NCHS 2013	0·94	0·85, 1·04	1·04	0·90, 1·19	0·95	0·79, 1·15
Social and community context						
Percent AI/AN alone, Census 2010‖						
Counties above median	0·89	0·80, 1·003	0·92	0·82, 1·04	0·90	0·73, 1·12

ACS, American Community Survey; FPL, federal poverty level; AI/AN, American Indian and Alaska Native; NCHS, National Centre for Health Statistics.

* *P* < 0·05.

† Data source: American Community Survey 2010–2014 5-year estimates.

‡ Data source: US Census Bureau 2000.

§ Data source: US Department of Agriculture Food Environment Atlas.

‖ Data source: US Census Bureau 2010.

** *P* < 0·01.

*** *P* < 0·001.

With respect to education and economic stability, persons who resided in counties with lower educational attainment (< high school) were more likely to be obese (OR = 1·20, 95 % CI (1·08, 1·34), *P* < 0·001). In stratified analyses, this association was seen in both subpopulations (lower-poverty counties: OR = 1·17, 95 % CI (1·05, 1·30), *P* < 0·01; higher-poverty counties: OR = 1·28, 95 % CI (1·06, 1·55), *P* < 0·05). People were significantly less likely to be obese if they lived in a county with lower access to a grocery store (OR = 0·87, 95 % CI (0·79, 0·97), *P* < 0·01). Stratified analyses indicated that the significant association between low grocery store access and less obesity remained only among those who lived in higher-poverty counties (OR = 0·77, 95 % CI (0·63, 0·94), *P* < 0·05).

When we added Medicare coverage among adults aged < 65 years into the multivariate model, findings were similar to those above except that adults with Medicare coverage who were aged < 65 years were more likely to be obese (see online Supplemental Table).

## Discussion

In the USA, more than one in three adults are obese. Excess body fat increases an individual’s risk for many health conditions, including type 2 diabetes, osteoarthritis and heart disease^(2)^. AI/AN are more likely to be obese than their non-Hispanic White counterparts^(5)^. Also, research has found that a larger percentage of AI/AN Medicare beneficiaries compared to Whites are classified as obese (34·7 % *v*. 28·6 %, respectively)^(26)^. Our study provides further insight into the social determinants of obesity in AI/AN adults aged ≥ 50 years. A recent systematic review of obesity risk factors for AI/AN^(10)^ only identified three studies since 2010 that had adult samples, all of them substantially smaller than the current study. Of these three studies, one of rural Alaska Yup’ik participants (*n* 488) found that higher levels of perceived stress were associated with higher BMI^(27)^. A second study of 459 American Indian adults residing on reservations in California found that a history of childhood verbal abuse was associated with higher BMI^(28)^. The third study in the same study population found a significant inverse association of household income and education with obesity^(29)^.

Few studies have examined the association of health care coverage with obesity in any racial or ethnic group. In our study, persons enrolled in Medicaid were less likely to be obese than those with only access to IHS services. While this association did not remain significant when we stratified by lower- and higher-poverty counties, likely due in part to smaller sample sizes, the OR remained similar. We also found that persons who had private health insurance were more likely to be obese; this association remained only for those residing in counties with lower poverty. Researchers have hypothesised that having health insurance disincentivises behaviours that guard against poor health^(30)^. Research using the Behavioral Risk Factor Surveillance System (BRFSS) data in persons aged 25 to 55 years found that having any health insurance was associated with increased BMI but not with the probability of obesity^(30)^. Greater understanding of the influence of Medicaid and private health insurance on obesity is needed among AI/AN. Our study population was users of IHS, whose funding is considered inadequate to meet population needs^(31)^. IHS and tribal health programmes obtain reimbursement from Medicaid, Medicare and private insurance coverage for provided services; patients with such coverage have greater financial access to other providers than those who do not.

Barriers to health care among AI/AN who are eligible for IHS include transportation and distance to services^(32)^. We found that those who must drive ≥ 30 min to their source for primary health care were more likely to be obese. In stratified analyses, this association only remained for those residing in higher-poverty counties. Analyses of BRFSS data found that people living in counties with a larger per capita number of primary care providers were 20 % less likely to be obese^(33)^. While IHS as well as individual tribes have encouraged and supported efforts to support lifestyles designed for weight loss and control, more research is warranted to better appreciate the contextual factors affecting obesity among AI/AN. That is, many tribes have developed their own programmes to address obesity in the community. Such efforts that are tailored for a specific tribal community often are more successful, although the efficacy of such programmes is unknown^(34)^.

Study participants who lived in counties with lower educational attainment among residents were more likely to be obese. A systematic review of the association between educational attainment and obesity concluded that this inverse association is more common in developed countries than developing countries^(35)^. More recent research has concluded that even with greater educational attainment and/or earnings, racial/ethnic minorities have not experienced the same benefits with respect to healthy body weight as Whites^(36)^. Further examination of the association of education and obesity is needed that takes into consideration the role of race/ethnicity. Only one of the three relevant studies among AI/AN measured the association between education and obesity, which was non-significant^(29)^. Although Hodge *et al*. (2011) did not find an association, materials and programmes geared to helping persons reduce weight need to take educational levels and health literacy of the targeted populations into consideration.

The shortcomings of the food environment in AI/AN communities have been well described^(37)^. Per the US Department of Agriculture, 74 % of persons residing on tribal lands live > 1 mile from a large grocery store or supermarket compared to 41 % of the general US population^(38)^. In our study population, low access to grocery stores was associated with a decreased likelihood of being obese. This finding is consistent with other research that has found a positive association between greater grocery store access and increased obesity rates^(39)^. When this relationship was examined in the two subpopulations, we found it to be significant only among AI/AN who lived in counties with higher poverty. Grocery stores are considered a healthy food source through offering fresh fruit and vegetables as well as a variety of meats. Yet, the body of research examining the association of grocery store access and weight-related outcomes has not established a clear picture. That is, two reviews determined that most research has not found an association between food outlet availability and obesity^(40,41)^ with some evidence, such as our results, of inverse associations between supermarket availability and obesity^(40)^. To date, there remains limited evidence for associations between local food environments and obesity. To better understand the role of grocery store access has on obesity among older AI/AN, it is suggested that a more comprehensive measurement of individuals’ usual food sources and access is used that captures the use of non-traditional food sources and food-related programmes.

Our study has several limitations worth acknowledgement. First, we excluded persons with diabetes in this study because of the obesity paradox in persons with diabetes, which could make findings on the association between social determinants and obesity among those with diabetes hard to interpret. That is, a literature review^(12)^ identified a large body of research that found being overweight or obese is associated with a lower mortality rate and better health outcomes in persons with diabetes. The phenomenon of obesity paradox has only been observed in studies relying on BMI rather than measurements of body fat or waist-to-hip ratio. Unfortunately, our data did not contain such measurements for us to examine. Moreover, confounding factors that have been theorised to play a role in this paradoxical association, such as smoking and fitness levels, were not available in our data. Therefore, we decided to focus on adults without diabetes in the current study. However, this precludes us from generalising our findings to those with diabetes, a prevalent chronic condition among AI/AN adults.

Furthermore, as our sample included only people who receive health care through the IHS, the study population is not representative of all AI/AN throughout the USA, and a sizable percentage of adults were excluded from the data set because they did not have a valid BMI measure in FY2013. While our data are not comparable to community-based surveys, the high prevalence of obesity among the IHS patients we examined emphasises the importance of research conducted in health care settings. The convenience sample may also limit the generalisability of the findings. Not all AI/AN can use IHS services as only members of federally recognised tribes and their descendants are eligible to receive direct care from the IHS. Also, our data are 9 years old, which can be considered a limitation given considering the ongoing efforts both at the federal and tribal level to decrease obesity. An additional limitation is that the data informing these analyses are cross-sectional, which prevent assessments of causality. Although these data allowed us to examine a large number of AI/AN, we did not have the level of detail that would have been available from medical record reviews or body composition changes. Increased age and fat redistribution leads to waist circumference and waist-to-hip ratio changes in older adults. Consequently, BMI may underestimate adiposity for ageing individuals^(42)^. Future studies will benefit from primary data collection efforts to allow for a more comprehensive assessment of body composition.

Despite these limitations, our current study has important strengths and contributes to the limited understanding of obesity among older AI/AN. This is the first large study with a geographically diverse AI/AN patient population to examine social determinants of obesity among older AI/AN who are at high risk for a number of obesity-related chronic conditions. Future studies should expand the inquiry into the social determinants of obesity in Native older adults. Prior research suggests a potential role for stress and childhood adverse events^(28,29)^. Social determinants of AI/AN nutritional health include historical trauma, boarding schools, adverse childhood experiences and food access^(6)^. As reflected in the ecological model proposed to understand childhood obesity among Aboriginal persons in Canada^(9)^, historical factors frame social determinants of health for AI/AN elders. For instance, a notable proportion of today’s elders had a negative boarding school experience^(43)^. Also, historically, foods available via the Food Distribution Program on Indian Reservations have included bleached flour, refined sugar, lard, sugar-sweetened beverages, corn syrup, processed meat and cheese^(44)^.

A construct of potential value to examine among AI/AN communities is known as ‘collective efficacy’^(45)^, which refers to informal social influence and social cohesion that reflects the willingness of community members to look out for each other and to get involved when there is perceived trouble^(46)^. With a sample of 3000 adults in Los Angeles County, research has found an association between increased collective efficacy and lower BMI, overweight risk, and overweight status^(47)^. Collective efficacy has been examined among AI/AN^(48)^ but not specifically within a tribal community. Given the AI/AN cultural value of community connectedness and emphasis on the collective^(49)^, examining how collective efficacy can be leveraged to support tribally based efforts to prevent and/or reduce obesity may be worthwhile.

Improving our understanding of social determinants of obesity among older AI/AN will only become more important. Obesity is associated with the leading causes of preventable death and a major driving force for the cardiometabolic epidemic in recent decades. Our findings highlight the need to investigate obesity among AI/AN, including longitudinal studies with a life course perspective to shed further light on the social determinants of obesity among older AI/AN.

## Supplementary material

For supplementary material accompanying this paper visit https://doi.org/10.1017/S1368980022000945. click here to view supplementary material
